# The Work Role Functioning Questionnaire v2.0 Showed Consistent Factor Structure Across Six Working Samples

**DOI:** 10.1007/s10926-017-9722-1

**Published:** 2017-09-09

**Authors:** Femke I. Abma, Ute Bültmann, Benjamin C. Amick III, Iris Arends, Heleen F. Dorland, Peter A. Flach, Jac J. L. van der Klink, Hardy A. van de Ven, Jakob Bue Bjørner

**Affiliations:** 1 Department of Health Sciences, Community and Occupational Medicine, University of Groningen, University Medical Center Groningen, Groningen, The Netherlands; 2 Robert Stempel College of Public Health & Social Work, Florida International University, Miami, FL USA; 3 Institute for Work & Health, Toronto, Canada; 4 Department Tranzo, Tilburg School of Social and Behavioral Sciences, Tilburg University, Tilburg, The Netherlands; 5 TNO, Leiden, The Netherlands; 6 National Research Centre for the Working Environment, Copenhagen, Denmark; 7 Optum, Lincoln, RI USA; 8 Department of Public Health, University of Copenhagen, Copenhagen, Denmark

**Keywords:** Confirmatory factor analyses, Work role functioning, Validity, Workers

## Abstract

*Objective* The Work Role Functioning Questionnaire v2.0 (WRFQ) is an outcome measure linking a persons’ health to the ability to meet work demands in the twenty-first century. We aimed to examine the construct validity of the WRFQ in a heterogeneous set of working samples in the Netherlands with mixed clinical conditions and job types to evaluate the comparability of the scale structure. *Methods* Confirmatory factor and multi-group analyses were conducted in six cross-sectional working samples (total N = 2433) to evaluate and compare a five-factor model structure of the WRFQ (work scheduling demands, output demands, physical demands, mental and social demands, and flexibility demands). Model fit indices were calculated based on RMSEA ≤ 0.08 and CFI ≥ 0.95. After fitting the five-factor model, the multidimensional structure of the instrument was evaluated across samples using a second order factor model. *Results* The factor structure was robust across samples and a multi-group model had adequate fit (RMSEA = 0.63, CFI = 0.972). In sample specific analyses, minor modifications were necessary in three samples (final RMSEA 0.055–0.080, final CFI between 0.955 and 0.989). Applying the previous first order specifications, a second order factor model had adequate fit in all samples. *Conclusion* A five-factor model of the WRFQ showed consistent structural validity across samples. A second order factor model showed adequate fit, but the second order factor loadings varied across samples. Therefore subscale scores are recommended to compare across different clinical and working samples.

## Introduction

Poor health is often associated with negative labor market outcomes including: sickness absence, low work performance and lower well-being [1–4]. Sickness absence due to chronic diseases constitutes a large economic burden to society [5]. Numerous studies show that lost productivity due to chronic disease constitutes an even larger burden [6, 7]. With the growing variety of measures to quantify the impact of health problems while at work, there remain few efforts to develop psychometrically sound instruments usable across different clinical or work populations. Multiple tools assessing lost productivity have been developed for specific diseases or work populations. From the perspective of building an evidence base there is an advantage to having a tool that is applicable across diseases and work populations (generic tools).

As work becomes more fragmented [8], it becomes important to assess the performance of generic work outcome tools across a range of working populations reflecting modern work. Similarly, research is needed to assess the validity of generic tools in different (sub-)clinical samples. It is often assumed that a generic tool is equally relevant in different clinical and subclinical samples. However, due to different work demands across occupations and different functional limitations due to clinical conditions, different association may be found across different samples using the same measurement tool. Comparative research regarding structural validity across multiple samples of heterogeneous clinical and working populations is important to allow valid group comparisons. Some research has already been done examining the comparative utility of the Job Content Questionnaire and the SF36 in different populations, but more is needed [9–13].

We take advantage of the opportunity to examine the construct validity of the Work Role Functioning Questionnaire v2.0 (WRFQ) [14] in a heterogeneous set of working samples with mixed clinical conditions and job types to assess the comparability of the scale structure. The WRFQ measures the ability of the worker to meet a range of job demands for the twenty-first century given a particular state of health. The WRFQ has been translated into over ten languages and can be used to measure the impact of health on functioning at work [15–22]. While different versions of the WRFQ exist, using both a four and a five factor structure, no study exist examining the factor structure of the WRFQ v2.0 using confirmatory factor analyses. Additionally, no evidence exists for the measurement equivalent across samples. Research on other widely used instruments such as the Job Content Questionnaire and the SF-36 has sought to demonstrate measurement equivalence across different occupational and clinical groups respectively. The WRFQ v2.0 integrates work and clinical conditions and thus to confidence the tool can be used in a range of occupational and clinical groups we are seeking to describe measurement equivalence in six different working and clinical samples in the Netherlands. Our two research questions are: (1) Is the structural validity of the WRFQ consistent across samples, and (2) Is the second order structure, a single work role functioning construct, consistent across samples?

## Methods

### Populations

The populations comprised six cross-sectional samples (N = 2433), described in Table 1. The samples were collected between 2010 and 2014 in various populations in the Netherlands. All participants were working at the moment of completing the WRFQ and were able to read, write and understand the Dutch language:

**Table 1:** Description of the six populations for gender, age, job type, health status, and work role functioning

N	CDP N = 229	CMDP N = 158	GWP N = 553	OIPP N = 154	SWP N = 1055	UWP N = 284
Age, M (SD)	50.8 (7.9)	42.3 (9.6)	45.1 (10.6)	53.7 (6.2)	44.0 (10.1)	45.6 (10.9)
Gender, N (%)						
Male	91 (1.3)	65 (41.1)	338 (70.2)	93 (60.4)	922 (87.4)	125 (44.0)
Female	135 (59.0)	93 (58.9)	165 (29.8)	52 (33.8)	117 (11.1)	159 (56.0)
Job type, N (%)						
Manual	23 (1.3)	^a^	156 (28.2)	0 (0)	256 (24.3)	^a^
Non manual	139 (60.7)	^a^	257 (46.5)	145 (100)	91 (8.6)	110 (61.3)
Mixed	64 (27.9)	^a^	5 (0.9)	0 (0)	638 (60.5)	^a^
Health status, N (%)						
Excellent	8 (3.5)	3 (1.9)	58 (10.5)	21 (13.6)	11 (1.0)	34 (12.0)
Very good	33 (14.4)	15 (9.5)	152 (27.5)	42 (27.3)	88 (8.3)	87 (30.6)
Good	129 (56.3)	92 (58.2)	281 (50.8)	71 (46.1)	291 (27.6)	127 (44.7)
Fair	53 (23.1)	42 (27.2)	55 (9.9)	10 (6.5)	91 (8.6)	30 (10.6)
Poor	3 (1.3)	2 (1.3)	3 (0.5)	1 (0.6)	4 (0.4)	5 (1.8)
Work role functioning total score, M (SD)	77.3 (17.6)	^b^	84.2 (15.8)	83.0 (12.6)	86.9 (13.7)	84.8 (14.4)
Work scheduling demands	77.3 (21.3)	65.3 (24.3)	83.0 (21.7)	80.9 (22.1)	86.6 (17.6)	83.9 (19.8)
Work output demands	74.6 (23.0)	64.7 (23.7)	81.0 (20.9)	76.6 (16.7)	84.7 (18.0)	79.8 (20.5)
Physical demands	83.7 (19.3)	90.5 (21.9)	87.1 (19.6)	94.0 (13.2)	89.0 (16.9)	91.6 (15.6)
Mental and social demands	75.4 (21.2)	64.1 (20.5)	85.2 (17.5)	85.8 (12.9)	87.5 (15.3)	85.0 (15.6)
Flexibility demands	78.4 (20.9)	^b^	84.0 (20.7)	80.3 (16.1)	87.4 (15.8)	85.1 (16.8)

Numbers might be lower/not add up to 100% due to missing

*CDP* cancer diagnosis population, *CMDP* common mental disorder population, *GWP* general working population, *OIPP* occupational and insurance physicians population, *SWP* shift worker population, *UWP* university workers population

^a^No information available

^b^Flexibility demands items missing, therefore no comparison score available

#### Cancer Diagnosis Population (CDP)

A sample diagnosed with cancer who returned to work in the last 3 months for at least 12 h per week. Data was collected in a longitudinal cohort study (WOLICA) in cancer survivors who recently returned to work after a cancer diagnosis [23, 24]. The sample is heterogeneous with respect to job type and cancer diagnoses (e.g. breast cancer, gastro-intestinal cancer, gynecological cancer, hematological cancer, urogenital cancer). Participants were recruited by their occupational physicians and received no incentive for participation. Patients with recurrent cancer and patients treated with palliative intent were excluded. More information about the sample can be found in the original publication. Baseline data was used.

#### Common Mental Disorder Population (CMDP)

A sample of workers who had partially or fully returned to work after a period of sick leave due to common mental disorders. Data was collected in a cluster-randomized controlled trial, the SHARP-at work study [25]. The sample is heterogeneous regarding job type and contains workers with various common mental health disorders (e.g. adjustment disorders, anxiety disorders, mild depression). Participants were recruited by participating occupational physicians and received no incentive for participation. Workers with a sickness absence spell >12 months, severe mental disorders (e.g. psychotic disorders, bipolar disorders or post-traumatic stress disorder), a previous sickness absence spell due to a CMD 3 months prior to the present sickness absent spell were excluded. Half of the participants received a relapse prevention intervention from their occupational physician, the other half received care as usual. Baseline data was used.

#### General Working Population (GWP)

A sample from the general working population, recruited from several companies and organizations in diverse work settings using multiple approaches. Data was collected in a study evaluating the measurement properties of the WRFQ v2.0 [14]. All participants were at work for at least 12 h per week and received no incentive for participation. The sample is heterogeneous across job types and health status, workers could also participate if they had no health problem. More information about the sample can be found in the original publication. Baseline data was used.

#### Occupational and Insurance Physicians Population (OIPP)

A sample of occupational and insurance physicians. Participants attending a 1-day conference were asked to complete a paper version of the WRFQ v2.0 during a plenary session. The sample is rather homogeneous regarding job type. Participants received no incentive for participation.

#### Shift Worker Population (SWP)

A sample of shift workers with regular shifts, unregularly shifts, on call workers, and workers on day shifts. Data was collected within the sampling frame of the ‘Shift Your Work’ study; a study about the effects of irregular night and shift work on health, work role functioning and social life [26, 27]. Regarding health status this is a heterogeneous sample. Participants received no incentive for participation. Baseline data was used.

#### University Workers Population (UWP)

A sample of university workers heterogeneous regarding job type (both academics and supporting staff) and health status. Data was collected in a cross-sectional study comparing workers who had returned to work after a sickness absence spell (>6 weeks) with workers who had no sickness absence in the past year [28]. Participants received no incentive for participation. Baseline data was used.

For each sample a data set was available containing at least:

- gender (male/female);
- self-rated health (excellent/ very good/good/ fair/poor) measured with the first question of the SF12 [29];
- job type (manual/non-manual/mixed, except in the UWP which only distinguishes between university vs. supporting staff);
- WRFQ v2.0 (except the CMDP, which did not contain the Flexibility demand items because data was collected prior to the development of these items).

Additionally, in the GWP and CDP information about number of chronic conditions were available.

### Work Role Functioning Questionnaire

The Work Role Functioning Questionnaire (WRFQ) measures the perceived difficulties in meeting work demands among employees given their physical health or emotional problems [14, 30]. The original WRFQ assessed five domains: work scheduling, output demands, physical demands, mental demands, and social demands. The second version of the WRFQ (v2.0) consists of 27 items, divided into four subscales: work scheduling and output demands (WSOD), physical demands (PD), mental and social demands (MSD), and flexibility demands (FD). Thus, compared to the original version, the subscales work scheduling demands and output demands have been combined, as have the mental demands and social demands subscales. Finally, a subscale on flexibility demands has been added. The recall period is 4 weeks and the response options range on a five-point scale from 0 = difficult all the time (100%), 1 = difficult most of the time, 2 = difficult half of the time (50%), 3 = difficult some of the time, 4 = difficult none of the time (0%). There is a response option ‘Does not apply to my job’. Subscale scores are calculated as the average of item scores multiplied with 25 to obtain scores between 0 and 100, with higher scores indicating better work role functioning. The scores on ‘Does not apply to my job’ were transformed to missing values. The score for a subscale was set to missing if 20% or more item scores were missing.

### Confirmatory Factor Analyses

Initial confirmatory factor analyses (CFA) were performed separately in each of the six data sets. Preliminary analyses were conducted exploring the factor structure in the various datasets using both exploratory and confirmatory analyses. The fit statistics for a four, five and six factor solution were explored and discussed to see if the proposed four factor structure should be used or if a better fit was found when the collapsed scales were separated [work scheduling and output demands (WSOD) and mental and social demands (MSD)]. Based on conceptual considerations and preliminary results, a five-factor model was found to fit best: work scheduling demands (WSD), output demands (OD), physical demands (PD), mental and social demands (MSD), and flexibility demands (FD) [14, 30], separating the work scheduling and output demands of the WRFQ v2.0 into two separate factors. This allowed comparison to the five factor structure of the original WRFQ.

The CFA analyses used methods for categorical data, analyzing polychoric correlation matrices using weighted least squares estimation with mean and variance adjustments (WLSMV) as implemented in the Mplus software [31]. Model fit was assessed by the following criteria: root mean square error of approximation (RMSEA) should be ≤0.08 and comparative fit index (CFI) should be ≥0.95 [32]. Further, a satisfactory model required that items should load >0.5 on the hypothesized factor and eventual cross-loadings on other factors should be <0.3. Revisions to a simple model structure were evaluated until satisfactory fit was achieved. The fit of the final model was compared to the fit of a five-factor model without modifications. The assumption of a common factor structure was tested through a multi-group CFA analysis in the five samples that used the full WRFQ v2.0 (GWP, CDP, OIPP, SWP, UWP). For this analysis, response categories had to be collapsed for several items (11, 12, 17, 18, 19, 22, 27), due to sparse responses in one or more subsamples. The multi-group model constrained factor loading and item thresholds to equality across samples but allowed factor means, variances, and co-variances, as well as residual correlations, to vary across samples.

After fitting the five-factor model, the multidimensional structure of the instrument was evaluated across working samples using a second order factor model. In addition to the previous specified fit criteria, loadings of the primary factors on the second order factor were expected to be >0.5 and similar across populations.

## Results

### Confirmatory Factor Analyses

Table 2 shows results of multifactor confirmatory factor analyses (CFA) performed separately in each of the six data sets. Three out of six models (for the CPD, CMDP and OIPP samples) specified two residual correlations [between ‘feel a sense of accomplishment in your work (item 9)’ and ‘feel you have done what you are capable of (item 10)’, and between ‘concentrate on your work (item 18)’ and ‘work without losing your train of thought (item 19)’]. Two further revisions in the form of cross-loadings were necessary to achieve adequate fit in all samples according the RMSEA and CFI statistics (see Table 2). First, in the CMDP sample, the output demands item ‘feel a sense of accomplishment in your work (item 9)’ loaded significantly on the ‘mental and social demands’ factor and had very low loading on the expected ‘output demands’ factor. Second, in the OIPP sample, the physical demands item ‘use hand-held tools or equipment (item 15)’ not only loaded on the ‘physical demands factor’, but also loaded 0.30 on the ‘mental and social demands’ factor. In all other respects, the factor structure was robust across samples and the factor loadings were high for all items—except for ‘lift, carry, or move objects >10 pounds (item 11)’ in the CMDP and UWP, and ‘process incoming information, for example e-mails, in time (item 25)’ in the GWP.

**Table 2:** Results of CFA multi-factor analyses

WRFQ 2.0	CPD					CMDP				GWP				
	WSD	OD	PD	MSD	FD	WSD	OD	PD	MSD	WSD	OD	PD	MSD	FD
WRFQ 1	0.70					0.77				0.78				
WRFQ 2	0.70					0.74				0.82				
WRFQ 3	0.71					0.62				0.73				
WRFQ 4	0.85					0.73				0.78				
WRFQ 5		0.80					0.89				0.89			
WRFQ 6		0.77					0.81				0.81			
WRFQ 7		0.85					0.83				0.82			
WRFQ 8		0.71					0.56				0.80			
WRFQ 9		0.72					**0.14**		**0.49**		0.77			
WRFQ 10		0.75					0.72				0.83			
WRFQ 11			0.51					**0.46**				0.55		
WRFQ 12			0.78					0.86				0.74		
WRFQ 13			0.71					0.93				0.77		
WRFQ 14			0.66					0.93				0.80		
WRFQ 15			0.67					0.75				0.85		
WRFQ 16				0.85					0.95				0.90	
WRFQ 17				0.92					0.83				0.91	
WRFQ 18				0.89					0.87				0.93	
WRFQ 19				0.88					0.78				0.87	
WRFQ 20				0.85					0.79				0.75	
WRFQ 21				0.75					0.71				0.57	
WRFQ 22				0.62					0.56				0.75	
WRFQ 23					0.75									0.81
WRFQ 24					0.66									0.80
WRFQ 25					0.63									**0.48**
WRFQ 26					0.80									0.82
WRFQ 27					0.85									0.90

	WSD	OD	PD	MSD	FD	WSD	OD	PD	MSD	WSD	OD	PD	MSD	FD
Factor correlations														
OD	0.75					0.63				0.79				
PD	0.52	0.50				0.35	0.34			0.42	0.33			
MSD	0.68	0.81	0.41			0.72	0.61	0.41		0.74	0.75	0.41		
FD	0.54	0.77	0.37	0.80						0.50	0.59	0.39	0.63	
Residual correlations														
WRFQ9/WRFQ10	0.26					0.35								
WRFQ18/WRFQ19	0.18					0.19								
Fit of revised 5F model	RMSEA = 0.071					RMSEA = 0.080								
	CFI = 0.973					CFI = 0.955								
Fit of simple 5F model	RMSEA = 0.081					RMSEA = 0.091				RMSEA = 0.072				
	CFI = 0.964					CFI = 0.941				CFI = 0.960				

WRFQ 2.0	OIPP					SWP					UWP				
	WSD	OD	PD	MSD	FD	WSD	OD	PD	MSD	FD	WSD	OD	PD	MSD	FD
WRFQ 1	0.91					0.78					0.84				
WRFQ 2	0.94					0.87					0.87				
WRFQ 3	0.77					0.66					0.75				
WRFQ 4	0.69					0.73					0.68				
WRFQ 5		0.84					0.85					0.85			
WRFQ 6		0.79					0.74					0.74			
WRFQ 7		0.88					0.82					0.81			
WRFQ 8		0.77					0.79					0.67			
WRFQ 9		0.63					0.76					0.80			
WRFQ 10		0.67					0.83					0.87			
WRFQ 11			0.76					0.59					**0.49**		
WRFQ 12			0.99					0.72					0.85		
WRFQ 13			0.88					0.74					0.82		
WRFQ 14			0.84					0.87					0.85		
WRFQ 15			0.67	**0.30**				0.84					0.92		
WRFQ 16				0.81					0.86					0.94	
WRFQ 17				0.97					0.93					0.91	
WRFQ 18				0.91					0.93					0.99	
WRFQ 19				0.92					0.88					0.98	
WRFQ 20				0.90					0.83					0.88	
WRFQ 21				0.82					0.70					0.85	
WRFQ 22				0.70					0.73					0.78	
WRFQ 23					0.80					0.97					0.88
WRFQ 24					0.81					0.97					0.74
WRFQ 25					0.83					0.94					0.81
WRFQ 26					0.82					0.97					0.88
WRFQ 27					0.82					0.98					0.92

	WSD	OD	PD	MSD	FD	WSD	OD	PD	MSD	FD	WSD	OD	PD	MSD	FD
Factor correlations															
OD	0.74					0.78					0.79				
PD	0.22	0.24				0.54	0.57				0.45	0.36			
MSD	0.63	0.70	0.25			0.58	0.69	0.55			0.63	0.71	0.51		
FD	0.57	0.66	0.03	0.81		0.20	0.31	0.21	0.41		0.53	0.68	0.55	0.88	
Residual correlations															
WRFQ9/WRFQ10	0.33														
WRFQ18/WRFQ19	0.10														
Fit of revised 5F model	RMSEA = 0.074														
	CFI = 0.962														
Fit of simple 5F model	RMSEA = 0.084					RMSEA = 0.055					RMSEA = 0.068				
	CFI = 0.950					CFI = 0.989					CFI = 0.981				

Bold numbers show loadings <0.5

*CDP* cancer diagnosis population, *CMDP* common mental disorder population, *GWP* general working population, *OIPP* occupational and insurance physicians population, *SWP* shift worker population, *UWP* university workers population, *WSD* work scheduling demands, *OD* output demands, *PD* physical demands, *MSD* mental and social demands, *FD* flexibility demands

Factor correlations were generally high, except for: (1) the ‘physical demands’ factor, which had low correlations with the other factors in all samples except the shift workers sample, (2) the ‘flexibility demands’ factor, which had low correlations with the other factors in the shift workers sample (but high correlation with other factors in all other samples).

A simple multi-group model for five samples (CDP, GWP, OIPP, SWP, UWP) without any specification of residual correlations or cross-loadings showed adequate overall fit (RMSEA = 0.063, CFI = 0.972).

The second order factor analyses showed high loading for all factors on the second order factor except for the physical demands factor, which had low loadings on the second order factor in three of the six samples (CMDP, GWP, OIPP). Additionally, low loadings on the second order factor were found for the flexibility demands factor in the shift worker population. When using the same model revisions on the first order level as applied in the previous analyses, all factor models had adequate fit according to the pre-specified criteria (Table 3). A second order multi-group model for five samples showed adequate overall fit, even when loadings on the second order factors were restricted to equality across most subsamples (RMSEA = 0.062, CFI = 0.972). However, the loading for ‘physical demands’ and ‘flexibility demands’ on the second order factor were significantly different in the SWP and UWP subsamples than in the other subsamples (Chisq = 43, DF = 4, p < 0.0001). In the SWP sample, standardized loadings were higher for ‘physical demands’ and lower for ‘flexibility demands’. In the UWP sample, standardized loadings were higher for both factors.

**Table 3:** Results of second order factor analyses

	CDP		CMDP		GWP		OIPP		SWP		UWP	
	Est	SE	Est	SE	Est	SE	Est	SE	Est	SE	Est	SE
WSD	0.76	0.03	0.85	0.04	0.86	0.02	0.76	0.05	0.79	0.02	0.75	0.03
OD	0.91	0.02	0.73	0.05	0.87	0.02	0.82	0.03	0.89	0.02	0.80	0.03
PD	0.50	0.07	**0.46**	**0.08**	**0.46**	**0.04**	**0.23**	**0.08**	0.66	0.03	0.55	0.05
MSD	0.91	0.02	0.85	0.04	0.87	0.02	0.89	0.03	0.79	0.02	0.92	0.02
FD	0.85	0.02	–	–	0.67	0.03	0.85	0.04	**0.39**	**0.03**	0.92	0.02
Fit statistics	RMSEA = 0.070 CFI = 0.973		RMSEA = 0.076 CFI = 0.959		RMSEA = 0.070 CFI = 0.962		RMSEA = 0.075 CFI = 0.960		RMSEA = 0.058 CFI = 0.987		RMSEA = 0.079 CFI = 0.974	

Bold numbers show loadings <0.5

*CDP* cancer diagnosis population, *CMDP* common mental disorder population, *GWP* general working population, *OIPP* occupational and insurance physicians population, *SWP* shift worker population, *UWP* university workers population, *WSD* work scheduling demands, *OD* output demands, *PD* physical demands, *MSD* mental and social demands, *FD* flexibility demands

## Discussion

The study aim was to explore the consistency of the structural validity and second order structure of the WRFQ v2.0 across six different working samples. Preliminary analyses found the best fit for a five-factor structure. This structure differed from the WRFQ v2.0 version [14] by separating the work scheduling demands from the output demands. This five-factor structure showed more resemblance to the original WRFQ structure [30]. The factorial structure appeared to be consistent across samples. While minor revisions were necessary to achieve good fit statistics in some samples, the WRFQ v2.0 instrument showed good structural validity for evaluating work role functioning in working samples with mixed clinical conditions and job types. To our knowledge this is the first time a comparison has been made for the factorial structure of a health-related work role functioning instrument across diverse working and clinical samples.

Three inconsistencies were identified: (1) In the CPD, CMDP and OIPP subsamples, two item pairs had residual correlations, suggesting that one item of each pair could be dropped from the questionnaire. (2) In the CMPD sample, item 9 'feeling a sense of accomplishment in your work’ loaded low (0.14) on the hypothesized ‘output demands’ factor and had higher loading (0.49) on the ‘mental and social demands’ factor. The item might be considered more of a mental challenge than an output demand in a sample with common mental disorders. (3) In the OIPP sample, item 15 ‘use handheld tools or equipment’ had a significant cross-loading on the mental and social demands factor but the strongest loading was still on the physical demands factor. In addition, item 11 (Lift, carry, or move objects at work weighing more than 10 pounds) had relative low factor loadings on the physical demands factor in all samples, indicating that this item is somehow measuring a different aspect of physical demands compared to the other items. Also, this item has many missing values because it is marked as ‘Not applicable’ by many participants. When aiming at item reduction, item 11 could be a candidate for deletion.

While a multi-group second order factor model had adequate overall fit, results suggest that the factor loadings vary between subsamples. In particular, loadings on the second order factor differed between the SWP and UWP sub-samples compared to the other subsamples for the physical demands (PD) and flexibility demands. The loading of PD on the second order factor was generally low, suggesting that the ability to meet physical work demands tends to be a separate aspect of work role functioning compared to the other included aspects. In separate analyses by sample, this was particularly the case for occupational and insurance physicians. An explanation could be that their work does not have (these) physical demands and, consequently, do not impact their work role functioning in the same way as the other demands.

When looking at the six samples, the heavily manual jobs are underrepresented. This underrepresentation might have influenced the results because it is often in the manual jobs that the physical demands are more present. Recent work on the factor structure of the Work Limitations Questionnaire, a closely related instrument measuring work limitations [33], also showed similar results regarding the physical demands subscale [34]. Additionally, the flexibility demands factor had low loadings on the second order factor in the shift worker sample. It might be that the items in the flexibility factor are less relevant for this heterogeneous sample containing multiple types of shift and day workers such as operators, police officers and technical support staff.

The results from the second order factor analyses suggest that the questionnaire may work differently in different samples. Especially the physical demands appear to behave differently across occupational samples. When comparing WRFQ v2.0 results across samples, we recommend using the subscale scores rather than the total score due to the different second order loadings in the various samples. In future research we should be aware of the implications of these differences when comparing across different job types and clinical samples.

A major strength if this study is the inclusion of six different samples across a variety of working samples. Unfortunately, due to the differences in data collection it was only possible to compare on an aggravated level (manual vs. non manual) and not on occupations level across datasets. Additionally, relatively few clinical groups were included, questioning the generalizability of the results to other conditions such as musculoskeletal conditions. Future research should focus on including more physically demanding jobs, multiple clinical condition groups, and samples across countries as the current study only included samples from one country. The results also point out directions for item reduction. Further advanced psychometric modelling, such as Item Response Theory and Differential Item Functioning, could be used to further refine the WRFQ and improve its practical application.

In conclusion, the WRFQ v2.0 shows consistent structural validity across samples. However, it is recommended to use the subscale scores to compare between different samples. The results are consistent for the second order structure except for physical demands and for flexibility demands in the shift work and university populations.
